# A Locked Nucleic Acid (LNA)-Based Real-Time PCR Assay for the Rapid Detection of Multiple Bacterial Antibiotic Resistance Genes Directly from Positive Blood Culture

**DOI:** 10.1371/journal.pone.0120464

**Published:** 2015-03-16

**Authors:** Lingxiang Zhu, Dingxia Shen, Qiming Zhou, Zexia Li, Xiangdong Fang, Quan-Zhen Li

**Affiliations:** 1 CAS Key Laboratory of Genome Sciences & Information, Beijing Institute of Genomics, Chinese Academy of Sciences, Beijing, China; 2 Department of Immunology, University of Texas Southwestern Medical Center at Dallas, Dallas, Texas, United States of America; 3 Department of Clinical Microbiology, General Hospital of People’s Liberation Army, Beijing, China; 4 State Key Laboratory of Mycology, Institute of Microbiology, Chinese Academy of Sciences, Beijing, China; Ghent University, BELGIUM

## Abstract

Bacterial strains resistant to various antibiotic drugs are frequently encountered in clinical infections, and the rapid identification of drug-resistant strains is highly essential for clinical treatment. We developed a locked nucleic acid (LNA)-based quantitative real-time PCR (LNA-qPCR) method for the rapid detection of 13 antibiotic resistance genes and successfully used it to distinguish drug-resistant bacterial strains from positive blood culture samples. A sequence-specific primer-probe set was designed, and the specificity of the assays was assessed using 27 ATCC bacterial strains and 77 negative blood culture samples. No cross-reaction was identified among bacterial strains and in negative samples, indicating 100% specificity. The sensitivity of the assays was determined by spiking each bacterial strain into negative blood samples, and the detection limit was 1–10 colony forming units (CFU) per reaction. The LNA-qPCR assays were first applied to 72 clinical bacterial isolates for the identification of known drug resistance genes, and the results were verified by the direct sequencing of PCR products. Finally, the LNA-qPCR assays were used for the detection in 47 positive blood culture samples, 19 of which (40.4%) were positive for antibiotic resistance genes, showing 91.5% consistency with phenotypic susceptibility results. In conclusion, LNA-qPCR is a reliable method for the rapid detection of bacterial antibiotic resistance genes and can be used as a supplement to phenotypic susceptibility testing for the early detection of antimicrobial resistance to allow the selection of appropriate antimicrobial treatment and to prevent the spread of resistant isolates.

## Introduction

The spread of drug-resistant bacterial strains has become a great threat to public health [1]. The mechanism of drug resistance is related with the acquisition of enzymes that inactivate antibiotic molecules or target gene mutation. A series of drug resistance genes have been identified, including *mec*A, *van*A, and *van*B in gram-positive bacteria [2–4] and extended-spectrum β-lactamase (ESBL) genes, plasmid-acquired AmpC lactamases (pAmpC) genes [5,6], and carbapenemase genes [7–11] in gram-negative bacteria. The rapid and accurate detection of drug resistance genes prior to treatment is very important for the selection of effective antibiotics to control infection.

The traditional method for detecting drug-resistant bacterial strains is antimicrobial susceptibility testing (AST). This method is based on bacterial cultivation, which requires 1–3 days for a susceptibility report and is also labor—intensive and time consuming. Many molecular methods, such as multiplex PCR, real-time PCR or microarray assay, have emerged recently for the detection of antimicrobial resistance genes, including *mec*A [12], *van*A or *van*B [13–15], ESBLs or pAmpC [16–22], and carbapenemase genes such as *bla*_KPC_, *bla*_NDM-1_, *bla*_VIM_ or *bla*_IMP_ [23–27]. Although some of these assays have been used in clinical detection and diagnosis [14,21,28], little has been reported about the simultaneous detection of multiple drug resistance genes directly from positive blood culture. In this study, we developed a locked nucleic acid (LNA)-based quantitative real-time PCR assay (LNA-qPCR) [29] for the rapid detection of thirteen antibiotic resistance genes that confer drug resistance in most of the common clinical bacterial strains.

## Materials and Methods

### Bacterial strains and specimens

The reference strains used in this study are listed in Table 1, including 13 strains harboring resistance genes detected in the PCR assay, 14 strains containing resistance genes not detected in the PCR assay, and 13 frequently encountered bacterial species in the clinical setting. Most of the reference strains were purchased from American Type Culture Collection (Rockville, Maryland, USA), and two strains were purchased from HPA Culture Collections (London, United Kingdom). Moreover, one *A*. *baumannii* AC 54/97 strain harboring IMP-2 and one *E*. *coli* DH5α strain harboring the *bla*_CTX-M-14_ gene were kind gifts from Prof. Gian M Rossolini. Three clinical strains, one each harboring *bla*_CTX-M-8_, *bla*_CTX-M-15_, or *bla*_CTX-M-16_, were kind gifts from Prof. James H. Jorgensen. Four *E*. *coli* strains, J53–2/pMG229, J53–2/pUD18, J53–2/pUD21, and C1 NalR/pAFF2, harboring the *bla*_SHV-2_, *bla*_SHV-3_, *bla*_SHV-4_, *bla*_SHV-5_ genes, respectively, were kind gifts from Prof. George Jacoby.

**Table 1:** Reference strains in this study.

Species	Strain No.	Drug resistance gene
*Staphylococcus aureus*	ATCC 43300	*mec*A
*Enterococcus faecium*	ATCC 51559	*van*A
*Enterococcus faecalis*	ATCC 51299	*van*B
*Citrobacter freundii*	ATCC 8090	chromosomal AmpC (*bla*_CMY-2_)
*Morganella morganii*	ATCC 25830	chromosomal AmpC (*bla*_DHA-1_)
*Escherichia coli*	DH5α/*bla*_CTX-M-14_	*bla*_CTX-M-14_
*Escherichia coli*	03–1814	*bla*_CTX-M-15_
*Acinetobacter baumannii*	AC-54/97	*bla*_IMP-2_
*Klebsiella pneumoniae*	ATCC BAA-1705	*bla*_KPC-2_
*Klebsiella pneumoniae*	ATCC BAA-2146	*bla*_NDM-1_
*Acinetobacter baumannii*	NCTC 13301	*bla*_OXA-23_
*Pseudomonas aeruginosa*	NCTC 13437	*bla*_VIM-10_
*Escherichia coli*	DH5α/T-OXA-58	*bla*_OXA-58_
*Escherichia coli*	ATCC 35218	*bla*_TEM-1_
*Klebsiella pneumoniae*	ATCC 13883	*bla*_SHV-1_
*Klebsiella pneumoniae*	ATCC 700603	*bla*_SHV-18_
*Escherichia coli*	J53–2/pMG229	*bla*_SHV-2_
*Escherichia coli*	J53–2/pUD18	*bla*_SHV-3_
*Escherichia coli*	J53–2/pUD21	*bla*_SHV-4_
*Escherichia coli*	C1 NalR/pAFF2	*bla*_SHV-5_
*Klebsiella oxytoca*	03–1921	*bla*_CTX-M-16_
*Klebsiella pneumoniae*	05–918	*bla*_CTX-M-8_
*Pseudomonas aeruginosa*	ATCC 27853	chromosomal AmpC
*Acinetobacter baumannii*	ATCC 19606	chromosomal AmpC
*Serratia marcescens*	ATCC 8100	chromosomal AmpC
*Enterobacter aerogenes*	ATCC 13048	chromosomal AmpC
*Enterobacter cloacae*	ATCC 13047	chromosomal AmpC
*Enterococcus casseliflavus*	ATCC 700327	
*Staphylococcus aureus*	ATCC 25923	
*Escherichia coli*	ATCC 25922	
*Klebsiella oxytoca*	ATCC 700324	
*Staphylococcus haemolyticus*	ATCC 29970	
*Staphylococcus epidermidis*	ATCC 12228	
*Enterococcus faecium*	ATCC 19434	
*Proteus mirabilis*	ATCC 7002	
*Proteus vulgaris*	ATCC 13315	
*Acinetobacter calcoaceticus*	ATCC 23055	
*Stenotrophomonas maltophilia*	ATCC 13637	
*Burkholderia cepacia*	ATCC 25416	
*Candida albicans*	ATCC 11006	

Thirty-seven non-repetitive, phenotypically resistant clinical isolates and thirty-five phenotypically susceptible clinical isolates were selected to verify the LNA-qPCR assays. These strains were collected from General Hospital of PLA (Beijing, China) and isolated from diverse sources from clinical patients during routine care, including strains of *Acinetobacter baumannii* (n = 4), *Escherichia coli* (n = 22), *Enterococcus faecalis* (n = 1), *E*. *faecium* (n = 4), *Klebsiella pneumoniae* (n = 6), *Pseudomonas aeruginosa* (n = 11), *Staphylococcus aureus* (n = 6), *S*. *epidermidis* (n = 12), *S*. *haemolyticus* (n = 5) and *S*. *hominis* (n = 1). All isolates were identified to the species level using the Vitek-2 system (bioMe´rieux, Marcy l’Etoile, France). Antimicrobial susceptibility testing (AST) was performed by the agar disk diffusion method according to Clinical and Laboratory Standards Institute (CLSI) guidelines [30]. Enterobacteriaceae isolates were screened for ESBL production by the CLSI phenotypic confirmatory method using disks containing 30 μg of cefotaxime and 30 μg of ceftazidime alone and in combination with 10 μg of clavulanate [30]. The minimal inhibitory concentrations (MICs) of several antibiotics, including cefotaxime, ceftazidime alone or in association with clavulanate (4 μg/ml), imipenem, oxacillin, and vancomycin, were determined for the clinical isolates by the agar dilution method with Muëller-Hinton agar (Tiantan biotechnology Co., Ltd., Beijing, China) using an inoculum of 10^4^ colony forming units (CFU) per spot [31].

In addition, 47 positive blood culture specimens were collected from General Hospital of PLA (Beijing, China) during the period of September 2011 to October 2011 for the detection of drug resistance genes using the LNA-qPCR assay. The samples from positive blood culture bottles were inoculated onto 5% sheep blood agar plates (BD Diagnostics, Sparks, MD) for primary isolation. Biochemical identification to the species level was performed using the Vitek-2 system (bioMe´rieux, France). Antimicrobial susceptibility testing was performed by the agar disk diffusion method according to CLSI guidelines [30]. *E*. *coli* ATCC25922, *K*. *pneumoniae* ATCC700603, *P*. *aeruginosa* ATCC27853, *S*. *aureus* ATCC25923 and ATCC 43300 were used as quality control strains for the AST experiments.

### Primer design and LNA TaqMan probe selection

The target-specific sequences of the desired antibiotic resistance genes were obtained from GenBank, and the representative sequences are listed in S1 Table. The primers and LNA probes were designed by multiple alignment analysis of the varieties using CLUSTAL W and are listed in Table 2. Primers were synthesized by Integrated DNA Technologies (Coralville, IA). The LNA probes were selected from the Universal ProbeLibrary (Roche Applied Science) based on online ProbeFinder Assay Design Software (http://qpcr.probefinder.com/) and were ordered from Roche Applied Science. All the nucleotides in the LNA probes are LNA nucleotides. The uniqueness of the primer sequences designed based on each target gene was evaluated with a BLAST search. Some primer and probe sets were designed to detect PCR products containing major substitutions for the identification of various β-lactamases, as follows: *bla*_CTX-M-1_-group ESBLs including *bla*_CTX-M-1_, *bla*_CTX-M-3_, *bla*_CTX-M-10–12_, *bla*_CTX-M-15_, *bla*_CTX-M-23_, *bla*_CTX-M-55_, *bla*_CTX-M-57_ and *bla*_CTX-M-79_; *bla*_CTX-M-9_-group ESBLs including *bla*_CTX-M-9_, *bla*_CTX-M-13–14_, *bla*_CTX-M-17–19_ and *bla*_CTX-M-21_; *bla*_CMY-2_ group pAmpCs including *bla*_CMY-2_, *bla*_CMY-3–7_, *bla*_CMY-12–18_, *bla*_CMY-20–41_, *bla*_CMY-43–44_ and *bla*_CMY-49_; *bla*_DHA-1_ group pAmpCs including *bla*_DHA-1–3_, *bla*_DHA-6_ and *bla*_DHA-7_; the *bla*_OXA-23_ group including *bla*_OXA-23_ and *bla*_OXA-27_; the *bla*_IMP_ group including *bla*_IMP-2_, *bla*_IMP-8_, *bla*_IMP-19–20_ and *bla*_IMP-24_; the *bla*_VIM_ group including *bla*_VIM-2–3_, *bla*_VIM-6_, *bla*_VIM-8–11_, *bla*_VIM-15–18_ and *bla*_VIM-23_; the *bla*_NDM-1_ group including *bla*_NDM-1_ and *bla*_NDM-2_; and the *bla*_KPC-2_ group including *bla*_KPC-2–10_.

**Table 2:** Sequences of primers and LNA probes.

Target Gene	LNA Probe No.	LNA Probe Sequence^a^	Primer Name	Primer Sequence (5′-3′)	Amplicon size (bp)
*bla*_CTX-M-1_ group	#119	ttggtggt	ctx-m-1-f	TGGGTTGTGGGGGATAAA	77
			ctx-m-1-r	CGATCTTTTGGCCAGATCAC	
*bla*_CTX-M-9_ group	#119	ttggtggt	ctx-m-9-f	TACCGACGTCGTGGACTG	79
			ctx-m-9-r	GGCCAGATCACCGCAATA	
*bla*_CMY-2_	#59	cagtggca	cmy-f	CTGGCCAGAACTGACAGGCA	68
			cmy-r	TAAGTGCAGCAGGCGGATA	
*bla*_DHA-1_	#59	cagtggca	dha-f	TGCGGATCTGCTGMAYTTCTA	96
			dha-r	GCACCAAACAGGCCGATA	
*bla*_KPC-2_	#40	gcctgctg	kpc-f	CTGTGCAGCTCATTCAAG	199
			kpc-r	CGGCGTTATCACTGTATTG	
*bla*_NDM-1_	#56	ggacagca	ndm-f	TATCACCGTTGGGATCGAC	84
			ndm-r	AGATTGCCGAGCGACTTG	
*bla*_OXA-23_	#56	ggacagca	oxa-23-f	CGTATTGGTTTCGGTAATGCT	70
			oxa-23-r	CCTTTAATGGTCCTACCAACCA	
*bla*_VIM-2_	#9	tggtgatg	vim-f	TCTACCCGTCCAATGGTCTC	66
			vim-r	CCCACGCTGTATCAATCAAA	
*bla*_OXA-58_	#9	tggtgatg	oxa-58-f	CAAGTGGTGGCATTTGCTT	94
			oxa-58-r	CCAACTTATCTAGCACATCTAAAGACA	
*bla*_IMP-2_	#119	ttggtggt	imp-f	TTAACGGTTGGGGTGTTGTT	70
			imp-r	TCAATCAGATAGGCGTCAGTGT	
*mec*A	#150	gaacagca	mecA-f	TTTAGACCGAAACAATGTGGAA	65
			mecA-r	TTGGAACGATGCCTATCTCA	
*van*A	#87	ggtggcag	vanA-f	CCCGCCTTTTGGGTTATTA	76
			vanA-r	CCGGCTTAACAAAAACAGGA	
*van*B	#82	cagaggag	vanB-f	TTATAACCGTTCCCGCAGAC	62
			vanB-r	TTTTGCCGTTTCCTGTATCC	
16S rRNA	#69	ggaggaag	16S-Uf	YAACGAGCGCAACCC	117
			16S-Ur	AAGGGSCATGATGAYTTGACG	

^a^: The sequence of the LNA probe was completely shown and all the nucleotides in the LNA probe were LNA nucleotides.

### DNA extraction

Bacterial DNA was extracted using a previously described rapid mechanical vortex method [29]. Briefly, 5 μl of positive blood culture sample was inoculated into 1 ml of TE (Tris-EDTA) buffer and mixed; 100 μl of the suspended cells was transferred into a lysis tube with glass beads (Sigma). The lysis tube was subjected to vortexing for 5 min at high speed, centrifuged briefly, and incubated at 95°C for 5 min to inactivate possible PCR inhibitors. The extracted DNA was stored at -20°C until use.

### LNA-qPCR assay and data analysis

A panel of 11 LNA-qPCR assays was set up for the parallel detection of multiple drug resistance genes, including two assays (*mec*A and *van*A/*van*B genes) for gram-positive bacteria, eight assays (*bla*_CTX-M-1_ */bla*_CTX-M-9_, *bla*_CMY-2_/*bla*_DHA-1_, *bla*_KPC-2_, *bla*_NDM-1_, *bla*_OXA-23_, *bla*_OXA-58_, *bla*_VIM_ and *bla*_IMP_ genes) for gram-negative bacteria, and one assay for the 16S rRNA gene as a positive external control. Positive (DNA mixtures with all the detected targets) and negative (sterilized water) PCR controls were prepared and included with each run.

The PCR reaction was optimized by testing different concentrations of each primer pair and the LNA probe, as well as the buffer component. Each PCR mixture (20 μl) contained 1× MasterMix (Applied Biosystems), with 5-carboxy-X-rhodamine succinimidyl ester (ROX) as an internal reference dye, deoxynucleoside triphosphates (including dUTP), and uracil-N-glycosylase. One microliter of lysate supernatant was added as a template. PCR was carried out using a 7900HT Fast Real-Time PCR System (Applied Biosystems) with two steps of amplification: an initial denaturation step at 94°C for 10 min, followed by 40 cycles of 94°C for 15 s and 60°C for 60 s.

The real-time PCR data were analyzed using ABI SDS2.3 software, and the average Ct (threshold cycle) value was generated for each sample. Based on our experiment with spike-in controls, a Ct value of 40 was set as a cut-off value for positivity. An assay was considered positive if the Ct value was less than 40. For each sample, 13 drug resistance genes and 16S rRNA gene were assayed in parallel. Samples with a Ct value below 40 for a resistance gene and the 16S rRNA gene were considered positive. Samples with a Ct value below 40 only for the 16S rRNA gene were also considered positive but without the detection of a resistance gene. Samples with a Ct value of 40 or higher for both a resistance gene and the 16S rRNA gene were considered negative. Samples with a Ct value below 40 for a resistance gene but with a Ct value of 40 or higher for the 16S rRNA gene were considered as uninterpretable.

The sensitivity of the LNA-qPCR assay was determined using serially diluted bacterial DNA from reference strains, which was spiked into pooled negative blood culture specimens. The concentration of microorganisms was determined and adjusted by standard counting of colony forming units (CFU) after plating suspensions on solid media. The spike-in concentrations were 5×10^5^, 5×10^4^, 5×10^3^, 500 and 50 CFU/ml (equivalent to 10^4^, 1000, 100, 10 and 1 CFU/reaction).

### Sequencing analysis

The presence of drug resistance genes was examined by regular PCR and further confirmed by DNA sequencing. The primers used for the PCR and DNA sequencing analysis are shown in S2 Table. The amplified PCR products were purified using the QIAquick PCR Purification Kit (QIAGEN Sciences, Maryland), and direct sequencing of the purified PCR products was performed using the BigDye Terminator 3.1 cycle sequencing kit (Applied Biosystems, Foster City, CA). Each sequence was then compared with known drug resistance gene sequences by multiple-sequence alignment using the BLAST program.

## Results

### Optimization and analytical sensitivity

The assays were designed for the parallel detection of 13 bacterial antibiotic resistance genes. After optimizing the amplification conditions for each assay, amplicons of the expected sizes were obtained by regular PCR from all reference strains, confirming the specificity of the primers (data not shown). The LNA-qPCR assay for each resistance gene was then optimized using different concentrations of the primers, probes, and BSA in the reaction mixture. The concentrations for the best detection limits were 200 nM for all LNA probes, 400 nM for all primer pairs (except for the *van*A gene, which was 600 nM), and 0.5% bovine serum albumin (BSA) as the reliever of PCR inhibition.

Linear-regression curves were generated for the detection of the serially diluted bacterial strains at concentrations from 10^4^ CFU to 1 or 10 CFU, and all strains showed a high correlation, with an R^2^ value of more than 0.97. The Ct and R^2^ values of each assay for the detection of different concentrations of bacterial strains are listed in S3 Table. All assays showed positive signals for the detection of bacterial cells at 10 CFU per reaction, with Ct value ranges of 16.0–35.9 and 16.6–34.5 for saline dilution experiments and negative blood culture spike-in experiments, respectively. When the bacterial concentration was diluted to 1 CFU per reaction, 8 assays (*bla*_CTX-M-1_ */bla*_CTX-M-9_, *bla*_CMY-2_/*bla*_DHA-1_, *bla*_KPC-2_, *bla*_NDM-1_, *bla*_OXA-58_, *bla*_IMP_, *mec*A, *van*A/*van*B) showed positive signals. The *bla*_VIM_ assay and *bla*_OXA-23_ assays did not show any positive signal in either the saline dilution or spike-in experiments. Therefore, the detection limits of all the assays were 1–10 CFU per reaction, which was similar for saline dilution and negative blood culture specimens. The Ct value was also comparable in the saline dilution and negative blood culture spike-in experiments, indicating that the blood culture medium has no significant effect on the analytical sensitivity of the real-time PCR assay. The three duplex PCR assays for *van*A/*van*B, *bla*_CTX-M-1_/*bla*_CTX-M-9_ and *bla*_CMY-2_/*bla*_DHA-1_ were able to detect 1 CFU per reaction, indicating the high amplification efficiency of these three duplex assays.

### The specificity of LNA-qPCR

The specificity of the LNA-qPCR assay was tested on the 14 resistant reference bacterial strains containing other resistance genes (Table 1). No cross-reaction with bacterial DNA among the different strains was observed, and all assays were positive for the 16S rRNA gene. The specificity and cross-reaction were further tested on 13 ATCC strains harboring no drug resistance genes but reported to be associated with nosocomial infections (Table 1). None of these 13 ATCC strains showed any cross-reaction with the ten resistance gene-specific LNA-qPCR assays, and all gave positive signals for the 16S rRNA gene assay. Furthermore, specificity was tested using another 77 frozen, archived negative blood culture specimens, and no false positive result was detected, indicating 100% specificity for the LNA-qPCR assays.

### Evaluation of LNA-qPCR using clinical isolates

To further verify the feasibility of the LNA-qPCR assay, 37 phenotypically resistant clinical isolates and 35 phenotypically susceptible clinical isolates were selected according to their phenotypic susceptibility profiles. DNA was extracted from a single colony picked from a solid medium plate. The results are shown in Table 3. All the isolates were positive for the external control (16S rRNA gene). All 35 susceptible isolates were negative for drug resistance genes by LNA-qPCR. Of the 37 drug-resistant clinical isolates, 27 were positive for one drug resistance gene, including *bla*_CTX-M-1/9_-type ESBL genes in gram-negative bacteria (10 *E*. *coli*, 2 *K*. *pneumoniae*), the *mec*A gene in gram-positive bacteria (n = 11), the *bla*_CMY-2_/*bla*_DHA-1_ gene (1 *E*. *coli*), the *bla*_OXA-23_ gene (2 *A*. *baumannii*), and the *bla*_VIM-2_ gene (1 *P*. *aeruginosa*). In addition, three strains harbored two different types of drug resistance genes, including one *E*. *coli* (*bla*_CTX-M-1/9_-type and *bla*_CMY-2_/*bla*_DHA-1_) and two *A*. *baumannii* strains (*bla*_CTX-M-1/9_-type and *bla*_OXA-23_; *bla*_OXA-23_ and *bla*_OXA-58_). The average Ct value range for these clinical isolates was 13.5–25.1. No *bla*_NDM-1_, *bla*_KPC-2_ or *bla*_IMP_-type carbapenemase genes or *van*A or *van*B gene were detected in the collected clinical isolates. All the LNA-qPCR results were confirmed by direct DNA sequencing, which showed that *bla*_CTX-M-79_ (4 strains), *bla*_CTX-M-15_ (4 strains), *bla*_CTX-M-55_ (2 strains), *bla*_CTX-M-3_ (1 strain), *bla*_CTX-M-9_ (2 strains) and *bla*_CTX-M-14_ (1 strain) accounted for the *bla*_CTX-M_ type ESBLs, and *bla*_CMY-2_/*bla*_DHA-1_ type pAmpC were *bla*_CMY-2_ and *bla*_DHA-1_ (1 strain for each gene). Overall, the concordance between LNA-qPCR results and AST results was 81.1% (30/37). However, 4 carbapenem-resistant *P*. *aeruginosa* strains, 1 cephalosporin-resistant *E*. *coli* strain, 1 cephalosporin-resistant *K*. *pneumoniae* strain, and 1 oxacillin-resistant *S*. *aureus* strain were negative by the LNA-qPCR assay.

**Table 3:** Real-time PCR testing of 37 clinically phenotypically resistant isolates.

Resistance genes detected by PCR	Clinical isolates	Phenotypic susceptibility^a^	DNA sequencing results
***bla***_**CMY-2**_ **/*bla***_**DHA-1**_	1 *E*. *coli*	CAZ^R^, CTX^R^, IMP^S^, MEM^S^	*bla*_DHA-1_
***bla***_**CTX-M-1**_ **/*bla***_**CTX-M-9**_	10 *E*. *coli*, 2 *K*. *pneumoniae*	CAZ^S^, CTX^R^, IMP^S^, MEM^S^	CTX-M-79 (n = 4), -15 (n = 3), -55 (n = 1), -3 (n = 1), CTX-M-9 (n = 2) and-14 (n = 1)
***bla***_**OXA-23**_	2 *A*. *baumannii*	CAZ^R^, IMP^R^, MEM^R^	*bla*_OXA-23_
***bla***_**VIM-2**_	1 *P*. *aeruginosa*	CAZ^R^, IMP^R^, MEM^R^	*bla*_VIM-2_
***mec*A**	2 *S*. *aureus*, 4 *S*. *haemolyticus*, 5 *S*. *epidermidis*	OXA^R^	*mec*A
***bla***_**CMY-2**_ **/*bla***_**DHA-1**_ **and *bla***_**CTX-M-1**_ **/*bla***_**CTX-M-9**_	1 *E*. *coli*	CAZ^R^, CTX^R^	*bla*_CMY-2_, CTX-M-15
***bla***_**CTX-M-1**_ **/*bla***_**CTX-M-9**_ **and *bla***_**OXA-23**_	1 *A*. *baumannii*	CAZ^R^, IMP^R^, MEM^R^	*bla*_OXA-23_, CTX-M-55
***bla***_**OXA-23**_ **and *bla***_**OXA-58**_	1 *A*. *baumannii*	CAZ^R^, IMP^R^, MEM^R^	*bla*_OXA-23_, *bla*_OXA-58_
**Not detected resistance gene**	4 *P*. *aeruginosa*	CAZ^R^, IMP^R^, MEM^R^	
	1 *E*. *coli*, 1 *K*. *pneumoniae*	CAZ^R^, CTX^R^, IMP^S^, MEM^S^	
	1 *S*. *aureus*	OXA^R^	
**Total**	37		

^a^: CAZ, ceftazidime; CTX, cefotaxime; IPM, imipenem; MEM, meropenem; OXA, oxacillin; VAN, vancomycin;

R: resistant;

S: susceptible.

### Performance test for clinically positive blood culture

Forty-seven positive blood culture samples were analyzed by LNA-qPCR for the parallel detection of 13 drug resistance gene. The external control (16S rRNA gene) was positive for all the blood culture samples, with a Ct value range of 16.2–25.9, indicating that the positive blood culture contained bacterial DNA. The concordance between LNA-qPCR and AST are shown in Table 4, S4 and S5 Tables. The average Ct value range of the LNA-qPCR assay for the positive blood culture samples was 18.2–30.3 (S4 Table).

**Table 4:** Analysis of positive blood culture samples by real-time PCR.

Antibiotic resistance gene	Blood culture Gram stain	AST resistant and PCR positive	AST susceptible and PCR negative	Only PCR positive	Only AST resistant	Concordance [%]
**ß-Lactamase genes** ^**a**^	Gram-negative rods	9 (8 Eco, 1 Kpn) ^**b**^	10(7 Eco, 3 Kpn)	1 (1 Kpn)	0	95.0 (19/20)
**Carbapenemase genes**	Gram-negative rods	0	17 (12 Eco, 5 Kpn)	0	3 (3 Eco)	85.0 (17/20)
***mec*A gene**	Gram-positive cocci	6 (1 Sau, 3 Sep, 2 Sha)	12 (3 Sau, 5 Sep, 1Sho, 2 Efm, 1 Efs)	3 (3 Sep)	0	85.7 (18/21)
***van*A / *van*B gene** ^**c**^	Gram-positive cocci	0	21 (2 Efm, 1 Efs, 18 Staph)	0	0	100 (21/21)
**Total**		15	60	4	3	91.5 (75/82)
**Sensitivity**		80.0%				
**Specificity**		90.0%				

Note: the positive blood culture samples containing *P*. *aeruginosa* are not included in Table 4.

^a^: ß-lactamase: *bla*_CTX-M-1_/*bla*_CTX-M-9_ ESBLs or *bla*_CMY-2_/*bla*_DHA-1_ pAmpC gene.

^b^: Eco, *Escherichia coli*; Kpn, *Klebsiella pneumoniae*; Pae: *Pseudomonas aeruginosa*; Sau, *Staphylococcus aureus*; Sep, *S*. *epidermidis*; Sha, *S*. *haemolyticus*; Sho, *S*. *hominis*, Efm, *Enterococcus faecium*; Efs, *E*. *faecalis*; and Staph, *Staphylococcus*.

^c^: *van*A and *van*B were not detected in the collected cultures.

Among 47 positive blood culture samples, 20 were identified as Enterobacteriaceae, including 15 *E*. *coli* and 5 *K*. *pneumoniae*. The LNA-qPCR results showed that nine samples (8 *E*. *coli* and 1 *K*. *pneumoniae*) contained *bla*_CTX-M-1/9_-type β-lactamase genes; one sample contained two types of β-lactamase genes (*bla*_CTX-M-1/9_-type and *bla*_CMY-2/DHA-1_-type), and other 10 samples did not contain any drug resistance genes. The LNA-qPCR results were 95% consistent with the results of phenotypic susceptibility for extended-spectrum cephalosporin resistance in Enterobacteriaceae, except that one *K*. *pneumoniae* strain harboring *bla*_CTX-M-15_ ESBL and *bla*_DHA-1_ pAmpC genes verified by DNA sequencing was susceptible to all the detected ß-lactam antibiotics, including ceftazidime and cefotaxime. For carbapenem resistance among Enterobacteriaceae, the concordance between real-time PCR results and traditional AST results was 85%. Three *E*. *coli* strains resistant to imipenem and/or meropenem were negative in LNA-qPCR (Table 4). In addition, six *P*. *aeruginosa* strains were identified from 47 positive blood culture samples. Among them, two *P*. *aeruginosa* strains resistant to ceftazidime, imipenem and meropenem were negative by LNA-qPCR.

Four *S*. *aureus* strains, 14 coagulase-negative staphylococci strains (including 11 *S*. *epidermidis*, 2 *S*. *haemolyticus* and 1 *S*. *hominis*), and three *Enterococcus* isolates (including two *E*. *faecium* and one *E*. *faecalis*) were subcultured and identified from 47 positive blood culture samples. Among them, nine samples (1 *S*. *aureus*, 6 *S*. *epidermidis* and 2 *S*. *haemolyticus*) contained *mec*A genes, though the other samples did not contain resistance genes, as detected by the LNA-qPCR assay. The LNA-qPCR results were 85.7% consistent with the susceptibility results for oxacillin resistance, except that 3 mecA-positive *S*. *epidermidis* samples were susceptible to oxacillin. Overall, the concordance between the LNA-qPCR assay and AST results was 91.5%. The sensitivity and specificity were 80% and 90.4%, respectively (Table 4).

## Discussion

The rapid identification of drug resistance genes directly from positive blood cultures is critical for the selection of appropriate antibiotics for the treatment of nosocomial infections, especially sepsis. Traditional phenotypic susceptibility results usually take an additional 1–2 days after a blood culture is reported to be positive. Some rapid susceptibility testing assays, such as shortened incubation of susceptibility tests by microfluidic system [32] and the use of functional mass spectrometry assays [33], have been used to improve the speed of AST results. However, a growth-based method may in some instances not detect resistant pathogens, e.g. methicillin resistance in *Staphylococcus* when using oxacillin, due to the known heterogeneous expression of the mecA gene [34–36].

In this study, we developed an LNA-based quantitative PCR assay (LNA-qPCR) to detect 13 clinically prevalent and important antibiotic resistance genes in bacteria: *mec*A, *van*A, *van*B and the *bla*_CTX-M-1_ group, *bla*_CTX-M-9_ group, *bla*_CMY-2_ group, *bla*_DHA-1_ group, *bla*_KPC-2_ group, *bla*_NDM-1_ group, *bla*_OXA-23_ group, *bla*_OXA-58_, *bla*_IMP_ group and *bla*_VIM_ group. The reason for the selection of these thirteen antibiotic resistance genes is that these genes are the most important contributing factors to resistance against antibiotics and are the most common in bacteria found in Chinese hospitals [37–43]. It should be noted that there are other drug resistance genes, as well as other mechanisms of antimicrobial resistance. In the future, the detection panel will be expanded for the extensive detection of additional clinically important antibiotic resistance genes, such as *bla*_OXA-48_, *bla*_SHV-2_, *bla*_SHV-5_, and *bla*_SHV-12_ ESBLs. It would be convenient to add resistance genes to the detection panel. However, each adaptation of the panel will require the multiplex LNA-qPCR technique to be optimized and validated again, before clinical application is possible.

The LNA probes used in the LNA-qPCR are short (8–9 nt) LNA-modified nucleotides labeled with fluorescein and can be used as hydrolysis probes in real-time PCR. The LNA-qPCR assay can be performed using extracted DNA obtained directly from positive blood bottles. The overall consistency between phenotypic susceptibility and the LNA-qPCR assay was 81.1% and 91.5% for clinically resistant isolates and positive blood cultures, respectively. The main discrepancy is with regard to carbapenem-resistant isolates, though no carbapenemase genes were detected, especially for *P*. *aeruginosa*. In total, only one of seven carbapenem-resistant *P*. *aeruginosa* strains contained *bla*_VIM-2_, as detected by the LNA-qPCR assay. Moreover, three carbapenem-resistant *E*. *coli* strains were also negative by LNA-qPCR. In addition to carbapenemase, a carbapenem-resistant phenotype in Enterobacteriaceae can be caused by decreased outer membrane permeability, which usually results in imipenem resistance, with low resistance or susceptibility to meropenem [44]. We observed two such *E*. *coli* strains. In the case of *P*. *aeruginosa*, reduced susceptibility to carbapenem can be due to non-carbapenemase resistance mechanisms, including the up-regulation of multidrug efflux pump systems such as MexAB-OprM or the loss of the outer membrane porin OprD [45].

Other discrepancies between AST and LNA-qPCR occurred mainly with Enterobacteriaceae, including two cephalosporin-resistant clinical isolates that were PCR negative, and one *E*. *coli* strain harboring *bla*_CTX-M-15_ and *bla*_DHA-1_ (confirmed by DNA sequencing) was susceptible to all the detected antibiotics. Considering the inducibility of the DHA-type enzyme, a greater risk of a poor therapeutic outcome based on its apparent susceptibility may occur. Moreover, three *mec*A PCR-positive *S*. *epidermidis* isolates were susceptible to oxacillin, perhaps due to the heterogeneous expression of the *mec*A gene [34].

We further simplified the assays for the detection of drug resistance in gram-negative bacteria by performing multiplex reactions as follows: one quadruplex reaction (*bla*_CTX-M-1_ */bla*_CTX-M-9_/*bla*_CMY-2_/*bla*_DHA-1_) and three duplex reactions (*bla*_OXA-23_/*bla*_OXA-58_, *bla*_IMP_/*bla*_VIM,_ *bla*_NDM-1_/*bla*_KPC-2_). The sensitivity and specificity was not affected by using reference strains, and these assays can accurately detect the target drug resistance genes (data not shown). By the evaluation of multiplex reactions using 47 positive blood culture samples, we showed that these multiplex PCR results were consistent with those of singleplex PCR. Our novel assay will greatly simplify technical operations and will be cost-efficient and easy to use in traditional clinical laboratories. PCR-positive results can provide clues for narrowing antimicrobial therapy, though isolates containing a drug resistance gene can be susceptible in AST [46–49] which points to the need to perform phenotypic testing. Although PCR-negative results are be insufficient to guide initial targeted antibiotic therapy due to undetected antibiotic resistance, such negative PCR results can support antibiotic choice for certain species if the latter is identified by a rapid method such as PCR [29] or mass spectrometry assays [50,51].

In summary, we developed an LNA-qPCR assay for the rapid (within 2 hours) detection of multiple bacterial antibiotic resistance genes directly from positive blood cultures. The LNA-qPCR assays have the potential to be used in addition to conventional microbiological methods for clinical susceptibility testing.

## Supporting Information

S1 Table The GenBank accession numbers used in the study. (DOCX)

S2 Table The primer sequences used for PCR and DNA sequencing. (DOCX)

S3 Table The analytical sensitivity of LNA-qPCR assay. (DOCX)

S4 Table The information of 47 positive blood culture samples. (DOCX)

S5 Table Drug susceptibility in clinical isolates from 47 positive blood culture samples. (XLSX)
