# Supplementary material for: A Locked Nucleic Acid (LNA)-Based Real-Time PCR Assay for the Rapid Detection of Multiple Bacterial Antibiotic Resistance Genes Directly from Positive Blood Culture
Source: PLoS One. 2015 Mar 16;10(3):e0120464. doi: 10.1371/journal.pone.0120464 (PMC4361058; doi:10.1371/journal.pone.0120464)
Supplement: S1 Table — (DOCX) [file pone.0120464.s001.docx]

**S1 Table. The GenBank accession numbers used in the study.**

| **Type** | **Accession No.** | **Protein encoded** |
| --- | --- | --- |
| CTX-M-1 group | X92506 | CTX-M-1 |
|  | Y10278 | CTX-M-3 |
|  | AF255298 | CTX-M-10 |
|  | AY005110 | CTX-M-11 |
|  | AF305837 | CTX-M-12 |
|  | DQ485309 | CTX-M-15 |
|  | AF488377 | CTX-M-23 |
|  | GQ456157 | CTX-M-55 |
|  | GU125662 | CTX-M-57 |
|  | HQ637574 | CTX-M-79 |
| CTX-M-9 group | AJ416345 | CTX-M-9 |
|  | AF252623 | CTX-M-13 |
|  | AJ972957 | CTX-M-14 |
|  | AY033516 | CTX-M-17 |
|  | AF325133 | CTX-M-18 |
|  | AF325134 | CTX-M-19 |
|  | AJ416346 | CTX-M-21 |
| CMY-2 group | DQ355981 | CMY-2 |
|  | Y16783 | CMY-3 |
|  | Y15130 | CMY-4 |
|  | Y17716 | CMY-5 |
|  | AJ011291 | CMY-6 |
|  | AJ011291 | CMY-7 |
|  | Y16785 | CMY-12 |
|  | AY339625 | CMY-13 |
|  | AJ555825 | CMY-14 |
|  | AJ555823 | CMY-15 |
|  | AJ781421 | CMY-16 |
|  | AY513266 | CMY-17 |
|  | AY743434 | CMY-18 |
|  | AY960293 | CMY-20 |
|  | DQ139328 | CMY-21 |
|  | DQ256079 | CMY-22 |
|  | DQ463751 | CMY-23 |
|  | EF415650 | CMY-24 |
|  | EU515249 | CMY-25 |
|  | AB300358 | CMY-26 |
|  | EU515250 | CMY-27 |
|  | EF561644 | CMY-28 |
|  | EF685371 | CMY-29 |
|  | EF685372 | CMY-30 |
|  | EF622224 | CMY-31 |
|  | EU496815 | CMY-32 |
|  | EU96816 | CMY-33 |
|  | EF394370 | CMY-34 |
|  | EF394371 | CMY-35 |
|  | EU331426 | CMY-36 |
|  | AB280919 | CMY-37 |
|  | AM931008 | CMY-38 |
|  | AB372224 | CMY-39 |
|  | EU515251 | CMY-40 |
|  | AB429270 | CMY-41 |
|  | FJ360626 | CMY-43 |
|  | FJ437066 | CMY-44 |
|  | GQ402541 | CMY-49 |
| DHA-1 group | Y16410 | DHA-1 |
|  | AF259520 | DHA-2 |
|  | AY494945 | DHA-3 |
|  | JF273491 | DHA-5 |
|  | HQ322612 | DHA-6 |
|  | HQ456945 | DHA-7 |
| IMP group | AJ243491 | IMP-2 |
|  | AF322577 | IMP-8 |
|  | EF118171 | IMP19 |
|  | AB196988 | IMP-20 |
|  | EF192154 | IMP-24 |
| VIM group | AF191564 | VIM-2 |
|  | AF300454 | VIM-3 |
|  | AY165025 | VIM-6 |
|  | AY524987 | VIM-8 |
|  | AY524988 | VIM-9 |
|  | AY524989 | VIM-10 |
|  | AY605049 | VIM-11 |
|  | EU419745 | VIM-15 |
|  | EU419746 | VIM-16 |
|  | EU118148 | VIM-17 |
|  | AM778091 | VIM-18 |
|  | GQ242167 | VIM-23 |
| NDM group | FN396876 | NDM-1 |
|  | JF703135 | NDM-2 |
| KPC group | AY034847 | KPC-2 |
|  | AF395881 | KPC-3 |
|  | AY700571 | KPC-4 |
|  | EU400222 | KPC-5 |
|  | EU555534 | KPC-6 |
|  | EU729727 | KPC-7 |
|  | FJ234412 | KPC-8 |
|  | FJ624872 | KPC-9 |
|  | GQ140348 | KPC-10 |
| OXA-23 group | AJ132105 | OXA-23 |
|  | AF201828 | OXA-27 |
| OXA-58 | AY570763 | OXA-58 |
| *mec*A | EF190335 | PBP2 |
| *van*A | FN424376 | VANA |
| *van*B | NC_005054 | VANB |
