# Supplementary material for: A Locked Nucleic Acid (LNA)-Based Real-Time PCR Assay for the Rapid Detection of Multiple Bacterial Antibiotic Resistance Genes Directly from Positive Blood Culture
Source: PLoS One. 2015 Mar 16;10(3):e0120464. doi: 10.1371/journal.pone.0120464 (PMC4361058; doi:10.1371/journal.pone.0120464)
Supplement: S2 Table — (DOCX) [file pone.0120464.s002.docx]

**S2 Table. The primer sequences used for PCR and DNA sequencing.**

| **Target Gene** | **Primer Name** | **Primer Sequence (5'-3')** | **Amplicon size (bps)** |
| --- | --- | --- | --- |
| *bla*_CTX-M-1_ group | ctx-m-1-f2 | TGGGTTGTGGGGGATAAA | 771 |
|  | ctx-m-1-r2 | CGATCTTTTGGCCAGATCAC |  |
| *bla*_CTX-M-9_ group | ctx-m-9-f2 | TGCAACGGATGATGTTCGCGG | 844 |
|  | ctx-m-9-r2 | ATGATTCTCGCCGCTGAAGCC |  |
| *bla*_CMY-2_ group | cmy-2-f2 | ATGATGAAAAAATCGTTATGC | 1146 |
|  | cmy-2-r2 | TTATTGCAGCTTTTCAAGAATG |  |
| *bla*_DHA-1_ group | dha-1-f2 | ATGAAAAAATCGTTATCTGCAAC | 1140 |
|  | dha-1-r2 | TTATTCCAGYGCACTCAAAATAG |  |
| *bla*_OXA-58_ group | oxa-58-f2 | CAAGTACAATTCCACAAGTG | 547 |
|  | oxa-58-r2 | CGCTCTACATACAACATCTC |  |
| *bla*_OXA-23_ group | oxa-23-f2 | TACTTGCTATGTGGTTGC | 768 |
|  | oxa-23-r2 | TCATTACGTATAGATGCCGG |  |
| *bla*_IMP_ group | imp-f2 | GAAGGYGTTTATGTTCATAC | 491 |
|  | imp-r2 | GGCCAAGCTTCTAWATTTGC |  |
| *bla*_VIM_ group | vim-f2 | GGTGAGTATCCGACAGTCA | 657 |
|  | vim-r2 | GAGCAAGTCTAGACCGCCCG |  |
| *bla*_KPC_ group | kpc-f2 | TCTGCTGTCTTGTCTCTCATGG | 613 |
|  | kpc-r2 | TCCCTTTAGCCAATCAACAAA |  |
| *mec*A | mecA-f2 | GATGGCTATCGTGTCACAATC | 352 |
|  | mecA-r2 | TGAGTTGAACCTGGTGAAGT |  |
| van*A* group | vanA-f2 | GAGCCGTTATACATTGGAAT | 901 |
|  | vanA-r2 | TCAAGCGGTCAATCAGTT |  |
| *van*B group | vanB-f2 | GGGGGTTGCTCAGAGGAGC | 999 |
|  | vanB-r2 | TCAAGCGGTCAATCAGTTC |  |
