# Supplementary material for: A Locked Nucleic Acid (LNA)-Based Real-Time PCR Assay for the Rapid Detection of Multiple Bacterial Antibiotic Resistance Genes Directly from Positive Blood Culture
Source: PLoS One. 2015 Mar 16;10(3):e0120464. doi: 10.1371/journal.pone.0120464 (PMC4361058; doi:10.1371/journal.pone.0120464)
Supplement: S3 Table — (DOCX) [file pone.0120464.s003.docx]

**S3 Table. The analytical sensitivity of LNA-qPCR assay.**

| **Strain** | DH5α/CTX-M-14 | 03-1814/CTX-M-15 | 8090/CMY-2 | 25830/DHA-1 | AC-54/97 | NCTC 13437 | BAA-1705 | BAA-2146 | NCTC 13301 | DH5α/T-OXA-58 | 43300 | 51559  /*van*A | 51299  /*van*B |
| --- | --- | --- | --- | --- | --- | --- | --- | --- | --- | --- | --- | --- | --- |
| **Assay** | *bla*_CTX-M-1_/*bla*_CTX-M-9_ | *bla*_CTX-M-1_/*bla*_CTX-M-9_ | *bla*_CMY-2_/*bla*_DHA-1_ | *bla*_CMY-2_/*bla*_DHA-1_ | *bla*_IMP_ | *bla*_VIM_ | *bla*_KPC-2_ | *bla*_NDM-1_ | *bla*_OXA-23_ | *bla*_OXA-58_ | *mec*A | *van*A/*van*B | *van*A/*van*B |
| **Saline dilution** |  |  |  |  |  |  |  |  |  |  |  |  |  |
| 10^4^ | 17.7 | 20.4 | 18.0 | 16.2 | 16.0 | 16.9 | 17.4 | 16.3 | 24.1 | 18.5 | 19.0 | 20.2 | 19.8 |
| 10^3^ | 21.3 | 24.2 | 21.4 | 19.7 | 19.2 | 20.0 | 22.5 | 21.0 | 27.3 | 22.4 | 22.2 | 24.2 | 21.9 |
| 100 | 24.7 | 27.4 | 24.5 | 23.2 | 22.7 | 23.4 | 26.0 | 25.3 | 31.3 | 26.6 | 27.3 | 27.8 | 25.9 |
| 10 | 29.8 | 31.6 | 28.8 | 27.0 | 27.8 | 26.3 | 32.4 | 30.9 | 35.9 | 28.3 | 30.5 | 30.6 | 30.2 |
| 1 | 33.9 | 35.9 | 35.6 | 33.1 | 31.6 | UD | 35.9 | 34.1 | UD | 32.1 | 33.8 | 34.8 | 33.9 |
| 0 | UD | UD | UD | UD | UD | UD | UD | UD | UD | UD | UD | UD | UD |
| R square value | 0.9944 | 0.9964 | 0.9716 | 0.9836 | 0.9935 | 0.9989 | 0.9925 | 0.9953 | 0.9944 | 0.9841 | 0.9932 | 0.9968 | 0.9891 |
| Detection limit (CFU/reaction) | 1 | 1 | 1 | 1 | 1 | 10 | 1 | 1 | 10 | 1 | 1 | 1 | 1 |
| **Spiked samples** |  |  |  |  |  |  |  |  |  |  |  |  |  |
| 10^4^ | 18.8 | 18.6 | 20.6 | 20.0 | 18.1 | 16.6 | 18.4 | 21.0 | 22.0 | 18.5 | 19.0 | 19.2 | 20.1 |
| 10^3^ | 22.8 | 22.0 | 23.4 | 22.3 | 22.2 | 20.3 | 22.5 | 24.0 | 26.3 | 20.4 | 22.2 | 22.2 | 23.9 |
| 100 | 26.2 | 25.9 | 26.8 | 26.4 | 24.8 | 23.9 | 26.2 | 26.7 | 30.7 | 24.2 | 25.9 | 25.7 | 26.9 |
| 10 | 30.8 | 30.4 | 30.9 | 30.6 | 29.6 | 26.0 | 28.9 | 31.2 | 34.1 | 27.8 | 29.6 | 30.0 | 31.3 |
| 1 | 33.9 | 33.4 | 33.6 | 33.1 | 33.9 | UD | 33.9 | 34.1 | UD | 31.6 | 33.1 | 34.0 | 34.5 |
| 0 | UD | UD | UD | UD | UD | UD | UD | UD | UD | UD | UD | UD | UD |
| R square value | 0.9975 | 0.9969 | 0.9962 | 0.9902 | 0.9927 | 0.9852 | 0.9934 | 0.9927 | 0.9974 | 0.9889 | 0.9996 | 0.9955 | 0.9967 |
| Detection limit (CFU/reaction) | 1 | 1 | 1 | 1 | 1 | 10 | 1 | 1 | 10 | 1 | 1 | 1 | 1 |

UD: undetermined
