# Supplementary material for: A Locked Nucleic Acid (LNA)-Based Real-Time PCR Assay for the Rapid Detection of Multiple Bacterial Antibiotic Resistance Genes Directly from Positive Blood Culture
Source: PLoS One. 2015 Mar 16;10(3):e0120464. doi: 10.1371/journal.pone.0120464 (PMC4361058; doi:10.1371/journal.pone.0120464)
Supplement: S4 Table — (DOCX) [file pone.0120464.s004.docx]

**S4 Table. The information of 47 positive blood culture samples.**

| Clinical No. | Species ID  by Vitek-2 | Ct of 16S rRNA gene | Ct of drug resistance gene(s) | Drug resistance genes by LNA-qPCR | Drug resistance genes by DNA sequencing |
| --- | --- | --- | --- | --- | --- |
| 2117 | *E. coli* | 22.9 |  |  |  |
| 2118 | *E. coli* | 21.8 |  |  |  |
| 4127 | *E. coli* | 21.0 | 27.2 | *bla*_CTX-M-1_/*bla*_CTX-M-9_ | *bla*_CTX-M-9_ |
| 7193 | *E. coli* | 23.0 | 25.4 | *bla*_CTX-M-1_/*bla*_CTX-M-9_ | *bla*_CTX-M-9_ |
| 8090 | *E. coli* | 24.6 | 24.0 | *bla*_CTX-M-1_/*bla*_CTX-M-9_ | *bla*_CTX-M-79_ |
| 13147 | *E. coli* | 19.0 | 22.7 | *bla*_CTX-M-1_/*bla*_CTX-M-9_ | *bla*_CTX-M-3_ |
| 15137 | *E. coli* | 23.6 |  |  |  |
| 19104 | *E. coli* | 18.9 |  |  |  |
| 19170 | *E. coli* | 21.6 | 22.9 | *bla*_CTX-M-1_/*bla*_CTX-M-9_ | *bla*_CTX-M-79_ |
| 20110 | *E. coli* | 20.6 | 21.2 | *bla*_CTX-M-1_/*bla*_CTX-M-9_ | *bla*_CTX-M-79_ |
| 20160 | *E. coli* | 19.9 | 21.9 | *bla*_CTX-M-1_/*bla*_CTX-M-9_ | *bla*_CTX-M79_ |
| 21151 | *E. coli* | 19.1 |  |  |  |
| 22160 | *E. coli* | 21.9 |  |  |  |
| 23211 | *E. coli* | 18.4 | 24.6 | *bla*_CTX-M-1_/*bla*_CTX-M-9_ | *bla*_CTX-M-9_ |
| 23212 | *E. coli* | 18.3 |  |  |  |
| 23169 | *E. faecalis* | 19.4 |  |  |  |
| 6119 | *E. faecium* | 24.6 |  |  |  |
| 14125 | *E. faecium* | 23.0 |  |  |  |
| 2099 | *K. pneumoniae* | 22.9 |  |  |  |
| 9130 | *K. pneumoniae* | 19.0 | 22.9 | *bla*_CTX-M-1_/*bla*_CTX-M-9_ | *bla*_CTX-M-15_ |
| 9206 | *K. pneumoniae* | 17.0 |  |  |  |
| 18045 | *K. pneumoniae* | 18.5 | 24.4/23.7 | *bla*_CTX-M-1_/*bla*_CTX-M-9,_ *bla*_CMY-2_*/bla*_DHA-1_ | *bla*_CTX-M-15_, *bla*_DHA-1_ |
| 30133 | *K. pneumoniae* | 17.3 |  |  |  |
| 13024 | *P. aeruginosa* | 20.0 |  |  |  |
| 14161 | *P. aeruginosa* | 17.0 |  |  |  |
| 21001 | *P. aeruginosa* | 18.0 |  |  |  |
| 30105 | *P. aeruginosa* | 24.4 |  |  |  |
| 20161 | *P. aeruginosa* | 20.2 |  |  |  |
| 22091 | *P. aeruginosa* | 20.5 |  |  |  |
| 9092 | *S. aureus* | 25.9 | 28.7 | *mec*A | *mec*A |
| 19167 | *S. aureus* | 19.8 |  |  |  |
| 19168 | *S. aureus* | 23.1 |  |  |  |
| 21114 | *S. aureus* | 25.9 |  |  |  |
| 7042 | *S. epidermidis* | 16.2 | 30.3 | *mec*A | *mec*A |
| 10139 | *S. epidermidis* | 21.3 |  |  |  |
| 12013 | *S. epidermidis* | 22.7 | 23.2 | *mec*A | *mec*A |
| 17167 | *S. epidermidis* | 19.2 |  |  |  |
| 19089 | *S. epidermidis* | 21.2 | 23.4 | *mec*A | *mec*A |
| 19090 | *S. epidermidis* | 22.1 | 21.6 | *mec*A | *mec*A |
| 20205 | *S. epidermidis* | 17.9 | 18.2 | *mec*A | *mec*A |
| 22139 | *S. epidermidis* | 23.7 |  |  |  |
| 23180 | *S. epidermidis* | 19.8 | 21.8 | *mec*A | *mec*A |
| 29084 | *S. epidermidis* | 17.2 |  |  |  |
| 30162 | *S. epidermidis* | 18.1 |  |  |  |
| 20201 | *S. haemolyticus* | 23.1 | 22.9 | *mec*A | *mec*A |
| 21008 | *S. haemolyticus* | 22.5 | 22.1 | *mec*A | *mec*A |
| 24103 | *S. hominis* | 18.6 |  |  |  |
